# Oral administration of *Moringa oleifera* leaf powder relieves oxidative stress, modulates mucosal immune response and cecal microbiota after exposure to heat stress in New Zealand White rabbits

**DOI:** 10.1186/s40104-021-00586-y

**Published:** 2021-05-12

**Authors:** Talat Bilal Yasoob, Defu Yu, Abdur Rauf Khalid, Zhen Zhang, Xiaofeng Zhu, Heba M. Saad, Suqin Hang

**Affiliations:** 1 National Center for International Research on Animal Gut Nutrition, Nanjing Agricultural University, No.1 WeiGang, Xuanwu region, Nanjing, 210095 Jiangsu China; 2 Laboratory of Gastrointestinal Microbiology, College of Animal Science and Technology, Nanjing Agricultural University, Nanjing, 210095 China; 3 Faculty of Agricultural Sciences, Ghazi University, Dera Ghazi Khan, 32200 Pakistan; 4 Department of Livestock and Poultry Production, Faculty of Veterinary Sciences, Bahauddin Zakariya University, Multan, 60000 Pakistan

**Keywords:** Cecal microbiota, Heat stress, *Moringa oleifera* leaf powder, Rabbits, Short chain fatty acids

## Abstract

**Background:**

Heat stress (HS) disrupts the gut barrier allowing the uptake of lipopolysaccharide (LPS) and leads to an inflammatory response and changes in gut microbiota composition. *Moringa oleifera* leaf powder (MOLP) has been proposed to combat HS, yet its alleviate role is currently under investigation. The current study investigated the effects of chronic HS and MOLP supplementation on changes in redox status and immune response of cecal mucosa along with alteration in cecal microbiota.

**Methods:**

A total of 21 young New Zealand White (NZW) rabbits (male) about 32 weeks old (mean body weight of 3318 ± 171 g) reared on a commercial pelleted diet were employed; divided into three groups (*n* = 7): control (CON, 25 °C), heat stress (HS, 35 °C for 7 h daily), and HS supplemented orally with MOLP (HSM, 35 °C) at 200 mg/kg body weight per day for 4 weeks.

**Results:**

The results demonstrated that MOLP supplementation increased organ index of cecal tissue compared with the HS group (*P* > 0.05). Levels of malonaldehyde (MDA) and activity of superoxide dismutase (SOD) as well as lactate dehydrogenase (LDH) were reduced in the cecal mucosa of the HSM group compared with the HS group. MOLP downregulated the contents of cecal mucosa LPS, several inflammatory markers (TNF-α/IL-1α/IL-1β), and myeloperoxidase (MPO) in the HSM group (*P* < 0.05). Secretory immunoglobulin A (SIgA) was increased in the HSM group compared with the HS group (*P* < 0.05). The transcriptome of cecal mucosa showed that MOLP reduced gene expression relative to several immune factors, including *IL-10*, *IFNG*, and *RLA*, whereas both HS and MOLP increased the gene expression of fat digestion and absorption pathway, including *APOA1*, *FABP1*, *FABP2*, *MTTP*, and *LOC100344166*, compared to the CON group (*P* < 0.001). At the phylum level, the relative abundance of Proteobacteria was increased by HS, while Actinobacteria was significantly increased by HSM compared to other groups (*P* < 0.05). At genus level, *Papillibacter* was higher in abundance in HSM groups compared to CON and HS groups (*P* < 0.05). Higher butyrate concentrations were observed in the HSM group than HS and CON groups (*P* < 0.05).

**Conclusion:**

In conclusion, HS in growing rabbits resulted in alteration of cecal microbiota at phyla level as well as increased oxidative stress and expression of mucosal inflammatory genes. Whereas, oral MOLP supplementation elevated the relative weight of cecum, affected their immunological and cecal micro-ecosystem function by improving antioxidant status and down-regulating mucosal tissue inflammatory response.

**Supplementary Information:**

The online version contains supplementary material available at 10.1186/s40104-021-00586-y.

## Background

The cecum of rabbits is proportionally the largest of any mammal. It is twice the length of the abdominal cavity and comprises 40–60% of the total volume of the gastrointestinal tract [1]. Moreover, 40% of the gastrointestinal digesta of rabbits is contained in its cecum [2], where the digesta is retained, mixed, and fermented by microbes [3]. As a cecum fermenter, rabbits possess a special re-ingestion system called cecotrophy. This system provides up to 30% of the rabbits’ daily N intake, which is mostly derived from cecal microbes [4, 5]. In addition, the short chain fatty acids (SCFAs) (acetic, formic, propionic, and butyric acids) derived from microbial fermentation in the cecum are actively absorbed through the cecal and colonic walls and utilized by the rabbit as energy sources [1]. Therefore, it is important for rabbits to create and sustain an optimal gut environment regardless of external environmental challenges.

Heat stress (HS) is one of the largest environmental impediments to efficient animal agriculture—especially rabbit husbandry, due to rabbits’ lack of functional sweat glands [6]. Although likely multifactorial, the nexus for many negative consequences of HS might be mediated by hyperthermia’s adverse effects on intestinal epithelial barrier [7]. Heat disrupts the tight junctions between enterocytes lining the intestinal tract and allows the uptake of intestinal pathogens and molecules, such as lipopolysaccharide (LPS) [8]. LPS is a Gram-negative bacterial cell wall component that can stimulate the immune system and, in large doses, cause harmful systemic inflammation [8]. HS suppresses different components of the immune system, thereby enhancing the susceptibility of an animal to various diseases and negatively affecting intestinal mucosa and microbiota composition [9, 10].

Feeding *Moringa oleifera* under normal conditions has been reported to improve the growth performance, FCR and health status in several livestock species when given at 5% or less of total dry matter intake [11]. Previously, we have also demonstrated the ability of *Moringa oleifera* leaf powder (MOLP) to improve growth performance, prevent intestinal morphological atrophy effectively, relieve oxidative damage, and downregulate mRNA expression of mucosal inflammatory genes in jejunum of the rabbits under HS conditions [12]. However, whether MOLP can contribute to homeostasis under HS in the cecum of New Zealand White (NZW) rabbits by modulating microbiome and transcriptome response is unknown. Therefore, the present study aimed to evaluate the potential role of MOLP under heat stress; and to explore the link between HS and the gut microbiome and association of HS with antioxidant and immune activity of cecal mucosa by using a high-throughput 16S rRNA sequence and transcriptome analysis.

## Methods

### Experimental design, animals, and housing

A total of 21 male NZW rabbits (32 ± 1 weeks of age) with similar body weight (3318 ± 171 g) were randomly assigned to each of the three treatment groups: control (CON), heat stress (HS), and HS with MOLP supplementation (HSM). Each group had seven replicates/rabbits and each rabbit was retained in individual cage. The nutrient composition of packaged MOLP obtained from commercial sources (Xishuangbanna, Yunnan Province, China) has been given (Supplementary Table 1). All the 21 rabbits were supplied with the pelleted feed ad libitum and fresh water round the clock with exception that HSM group received oral MOLP supplementation administered as gavage syringe at 200 mg/kg body weight once daily in the morning throughout the 28-day experiment. All rabbits in the CON group were maintained at 25 °C throughout the experiment. However, rabbits in the HS and HSM groups underwent thermal stress in thermostatically controlled rooms at 35 ± 1 °C for 7 h daily (09:00–16:00). The temperature of the heat stress houses was brought down to 25 °C for the remaining 17 h of the day after the 7 h of heat wave. Body weight of each rabbit was recorded twice a week and at the end of experiment for the determination of average daily weight gain (ADG). The feed consumption per cage was recorded daily to calculate average daily feed intake (ADFI). Later FCR was calculated by using data of ADG and ADFI.

### Sample collection and determination of cecal organ index

The samples were collected towards the end of trial. At day 29, final weighing of rabbits was done after fasting for 6 h. All 21 rabbits were slaughtered between 7:00 and 10:00 in a blinded procedure. The rabbits were humanely slaughtered according to the method described by Nakyinsige et al. [13] by severing carotid artery, jugular vein, trachea, esophagus, and vagus nerve. Their abdomens were incised and after immediate removal of cecum, the weights were measured. Organ indexes were expressed relative to body weight (g of organ per kg of body weight). Next, the cecal digesta samples and the cecal mucosa samples (after digesta removed and tissue rinsed with 0.9% NaCl) were collected into 2 mL Eppendorf tubes and frozen immediately in liquid nitrogen (− 80 °C). The mucosa tissue samples remained in liquid nitrogen while the cecal digesta samples were later kept at − 20 °C until further analysis.

### Oxidative stress level in cecal mucosa

Catalase (CAT) in the mucosal tissue of the cecum was measured using an ELISA kit (Angle Gene Biotechnology, Nanjing, China), whereas superoxide dismutase (SOD), glutathione S-transferase (GST), malonaldehyde (MDA), and lactate dehydrogenase (LDH) were measured using another ELISA kits (Nanjing Jiangchang Biotechnology, Nanjing, China) according to the manufacturer’s instructions.

### The level of inflammatory markers in cecal mucosa

The LPS, secretory immunoglobulin A (SIgA), tumor necrosis factor alpha (TNF-α), interleukin (IL)-1α, IL-1β and IL-6, and Myeloperoxidase activity (MPO) in the mucosal tissue of the cecum were measured using an ELISA kit (Angle Gene Biotechnology, Nanjing, China).

### Transcriptome analysis

To balance the cost and to select appropriate number of samples required for transcriptome analysis [14], total RNA from 9 cecal mucosa samples was extracted using the TRIZOL reagent (TaKaRa, Dalian, China). After extraction, the quality of the RNA was assessed by Nanodrop2000 (concentration and OD260/280 detection), agarose gel electrophoresis (RNA integrity detection), and Agilent2100 (RIN value measurement). Only high-quality total RNA samples (≥ 5 μg; ≥ 200 ng/μL; OD260/280 = 1.8 – 2.2) were used to construct sequencing libraries. The extracted samples were processed individually and later used to construct cDNA library following the protocol specified by the Truseq™ RNA sample prep kit (Illumina, San Diego, USA). Later, mRNA isolation and fragmentation (random fragments of 200 bp) were accomplished via Oligo (dT) and metal ions, respectively. mRNA fragments were transcribed into first-strand cDNA using reverse transcriptase and random primers, followed by second-strand cDNA synthesis. The double-stranded cDNA was further performed with end repaired, A base tailed, and indexed adapters were ligated. Then, the ligation product was amplified through polymerase chain reaction (PCR), and Illumina sequencing was performed by Biozerone, Shanghai, China. After an Illumina PE library was constructed, an Illumina platform was adopted to complete the 2 × 150 bp sequencing. Primary sequencing data (raw reads) were then subjected to quality control (QC). Tools such as SeqPrep (https://github.com/jstjohn/SeqPrep) and Sickle (https://github.com/najoshi/sickle) were also used. After obtaining clean reads, TopHat software (default parameters) was applied to align RNA-Seq reads to mammalian-sized genomes using the ultra-high-throughput short read aligner Bowtie, after which the mapping results were analyzed to identify splice junctions between exons.

A user-friendly software package, RSEM (http://www.biomedsearch.com/nih/RSEM-accurate-transcript-quantification-from/21816040.html), was used for quantifying gene and isoform abundances from single-end or paired-end RNA-Seq data. After sequencing, the expression level of each sequence library was standardized as FPKM, and the most differentially expressed genes (DEGs) were selected using EdgeR or deseq2 for further analysis.

### Microbiome analysis

The number of animals used in our study for microbiota analysis were decided keeping in view the economic concerns and on the basis of number of animals selected in previous studies [15–18]. Total genomic DNA of cecal microbiota was extracted from all the 21 samples with 0.3 g of homogenized cecal digesta samples using a bead-beating and phenol-chloroform method [19]. After determining the purity and concentration of DNA, it was finally diluted (1.0 ng/mL). The diluted DNA was used to amplify the V3-V4 hypervariable regions in the 16S rRNA gene amplicons with barcoded primers (341F: 5′-CCTAYGGGRBGCASCAG-3′, 806R: 5′-GGACTACNNGGGTATCTAAT-3′), which is related to the lowest taxonomic assignment error rate for 16S rRNA MiSeq sequencing [20]. PCR was performed in triplicate. Amplicons were extracted from 2% agarose gels and purified using the AxyPrep DNA Gel extraction kit (Axygen Biosciences, Union City, CA, USA) according to the manufacturer’s instructions, after which the amplicons were quantified using QuantiFluor-ST (Promega, Madison, WI, USA). The pooled DNA product was used to construct the Illumina Pair-End library following Illumina’s genomic DNA library preparation procedure. Purified amplicons were pooled in an equimolar and paired-end sequence (2 × 250) on an Illumina Miseq platform (Biozeron Biotechnology, Shanghai, China).

Raw fastq files were trimmed and chimeric sequences were identified and removed from all samples using UCHIME to reduce noise and to obtain Clean Reads. FLASH [21] was used to merge the paired-end reads generated from the DNA fragments. Non-assembled reads were discarded. The Clean Reads were then clustered as operational taxonomic units (OTUs) by scripts of the UPARSE software (version 7.1, http:// drive5.com/uparse/) with a 97% similarity threshold. The phylogenetic affiliation of each 16S rRNA gene sequence was analyzed by a Ribosomal Database Project Classifier (RRID:SCR_006633) against the Silva (SSU115) 16S rRNA database (RRID:SCR_006423) using a confidence threshold of 70% [22].

### Short chain fatty acid (SCFA) analysis

The cecal contents (stored at − 20 °C) from 21 samples were used for analysis of SCFAs using gas chromatography (GC2010 Plus, Shimadzu, Japan) as described previously [23]. Briefly, cecal digesta samples (50 mg) were suspended in 250 μL of ddH_2_O and were centrifuged at 12,000 × *g* for 5 min in a microcentrifuge (Microfuge 22R, Beckman Coulter, CA, USA). The SCFA analysis was carried out on the supernatants (200 μL), with crotonic acid as an internal standard. A flame ionization detector and a capillary column (Agilent Technologies, HP-INNOWax, 30 m × 0.25 mm × 0.25 μm, CA, USA) were used, with an injector/detector temperature of 180 °C/180 °C, a column temperature of 130 °C, and a gas flow rate of 30 mL/min.

### Statistical analysis

**Fig. 1:**
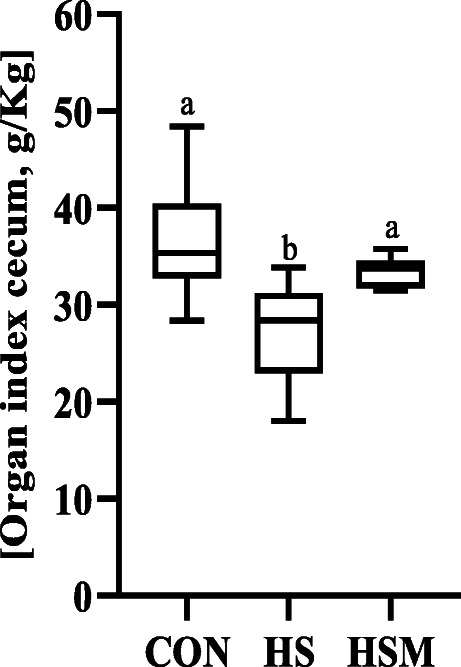
Effect of HS and *Moringa oleifera* leaf powder (MOLP) supplementation on organ index of cecum (*n* = 7 per group). MOLP: *Moringa oleifera* leaf powder; CON: control; HS: heat stress; HSM: heat stress with MOLP supplementation. All data is shown as mean values ± standard error of mean (SEM). ^a,b^ Means within groups (columns) with different superscript letters are significantly different (*P <* 0.05)

**Fig. 2:**
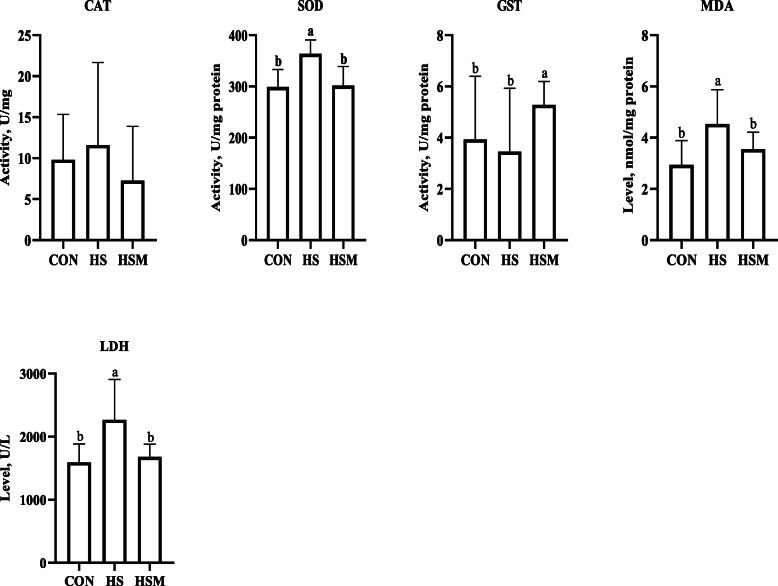
Effect of HS and MOLP supplementation on biochemical traits of cecal mucosa in NZW rabbits (*n* = 7 per group). MOLP: *Moringa oleifera* leaf powder; CON: control; HS: heat stress; HSM: heat stress with MOLP supplementation; CAT: catalase; SOD: superoxide dismutase; GST: glutathione S-transferase; MDA: malonaldehyde; LDH: lactate dehydrogenase. All data is shown as mean values ± standard error of the mean (SEM). ^a,b^ Means within groups (columns) with different superscript letters are significantly different (*P <* 0.05)

**Fig. 3:**
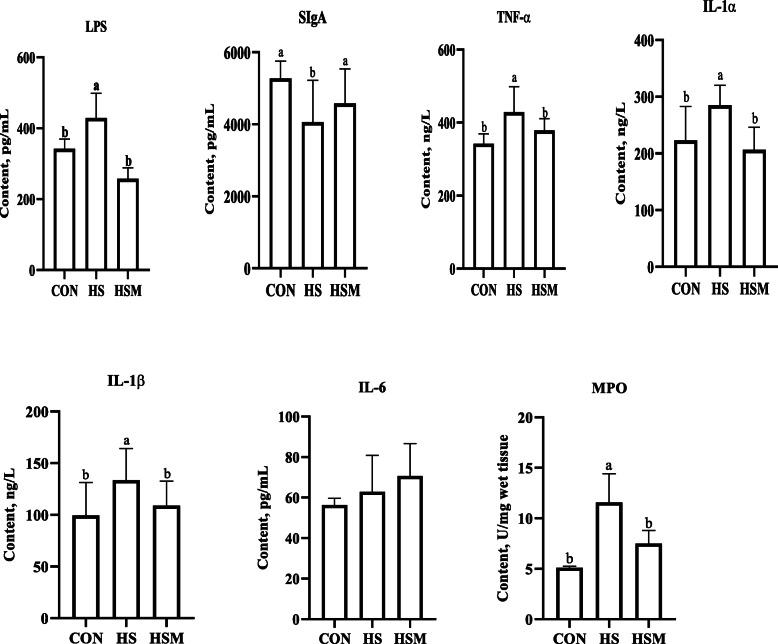
Effect of HS and MOLP supplementation on LPS, SIgA and markers of inflammation in cecal mucosa of NZW rabbits (*n* = 7 per group). MOLP: *Moringa oleifera* leaf powder; CON: control; HS: heat stress; HSM: heat stress with MOLP supplementation; LPS: lipopolysaccharide; sIgA: secretory immunoglobulin A; TNF-α: tumor necrosis factor alpha; IL-1α: interleukin-1α; IL-1β: interleukin-1β; IL-6: interleukin-6; MPO: myeloperoxidase. All data is shown as mean values ± standard error of the mean (SEM). ^a,b^ Means within groups (columns) with different superscript letters are significantly different (*P <* 0.05)

To determine the effect of heat stress and MOLP supplementation, the data for biochemical traits such as anti-oxidative contents and inflammatory markers of cecal mucosa were analyzed by One-way ANOVA using SPSS 21.0 (SPSS Inc., Chicago, IL, USA) software with replicates as experiment units. The results are presented as mean ± standard error of mean (SEM). Significant differences were at *P* < 0.05. Significant differences due to treatments were separated by Student Newman Keuls multiple range test. Figures 1, 2 and 3 were constructed using the GraphPad Prism (version 8, San Diego, CA, USA).

For transcriptomics data analysis, the significance levels of the transcripts were measured using the FDR (False Discovery Rate) control method to rationalize the *P*-value. The screening criterion to select the differential expressed genes between groups was used when threshold fold change |log_2_FC| ≥ = 1 & FDR < 0.05 [24, 25] Goatools (https://github.com/tanghaibao/GOatools) was exploited to identify statistically significantly enriched GO term using Fisher’s exact test. KOBAS 2.0 (http://kobas.cbi.pku.edu.cn/home.do) was used to identify a statistically significant enriched pathway using Fisher’s exact test.

For the analysis of microbial data, the community richness estimator (Chao and ACE), diversity indices (Shannon and Simpson), and Good’s coverage as well as relative abundance of phylum and genera among groups were compared using the nonparametric Kruskul-Wallis H test in SPSS 21. Principal component analysis (PCA) was used to compare groups of samples based on unweighted UniFrac distance metrics [26]. Whereas, analysis of LEfSe was carried out using online tool (https://huttenhower.sph.harvard.edu/galaxy/).

## Results

### MOLP supplementation improved growth parameters and organ index of cecum under HS

The data on growth performance has been given in Supplementary Table 2. Briefly, MOLP supplementation improved growth parameters such as ADG (25.8%, *P* < 0.05), ADFI (14.6%, *P* < 0.05) and FCR (12.4%, *P* < 0.05) compared to HS rabbits. The organ index of cecum to evaluate the effects of HS and *Moringa *supplementation at the end of trial were also recorded. Compared with the CON group, HS had profoundly reduced organ index of the cecum, whereas MOLP had improved organ index than that of HS group (Fig. 1**,** *P <* 0.05).

### Effect of HS and MOLP supplementation on cecal oxidative stress

Further, the effect of HS and MOLP on cecal mucosa levels of oxidation and anti-oxidation status was explored. The results showed that MDA levels and SOD activity in cecal mucosa were notably higher in the HS group (Fig. 2, *P* < 0.05). Activity of Catalase and GST did not differ among the groups. HS significantly elevated LDH activity compared to the CON group, whereas MOLP reduced it (Fig. 2, *P* < 0.05).

### Effect of HS and MOLP supplementation on markers of cecal mucosa inflammation

LPS content was significantly higher in the HS group compared with the CON group, whereas, LPS was restored to the normal level in the HSM group **(**Fig. 3, *P* < 0.05). SIgA was significantly lower in the HS group as compared to CON group and HSM groups (Fig. 3, *P* < 0.05). Inflammatory levels of TNF-α, IL-1α, and IL-1β were also significantly higher in the HS group compared with CON and HSM groups (*P* < 0.05), while they did not differ between the CON and HSM groups (*P* > 0.05). Myeloperoxidase (MPO) activity decreased significantly in the HSM group compared to HS group (*P* < 0.05). The contents of IL-6 did not differ significantly among the groups (*P >* 0.05).

### Transcriptome analysis of cecal mucosa

**Fig. 4:**
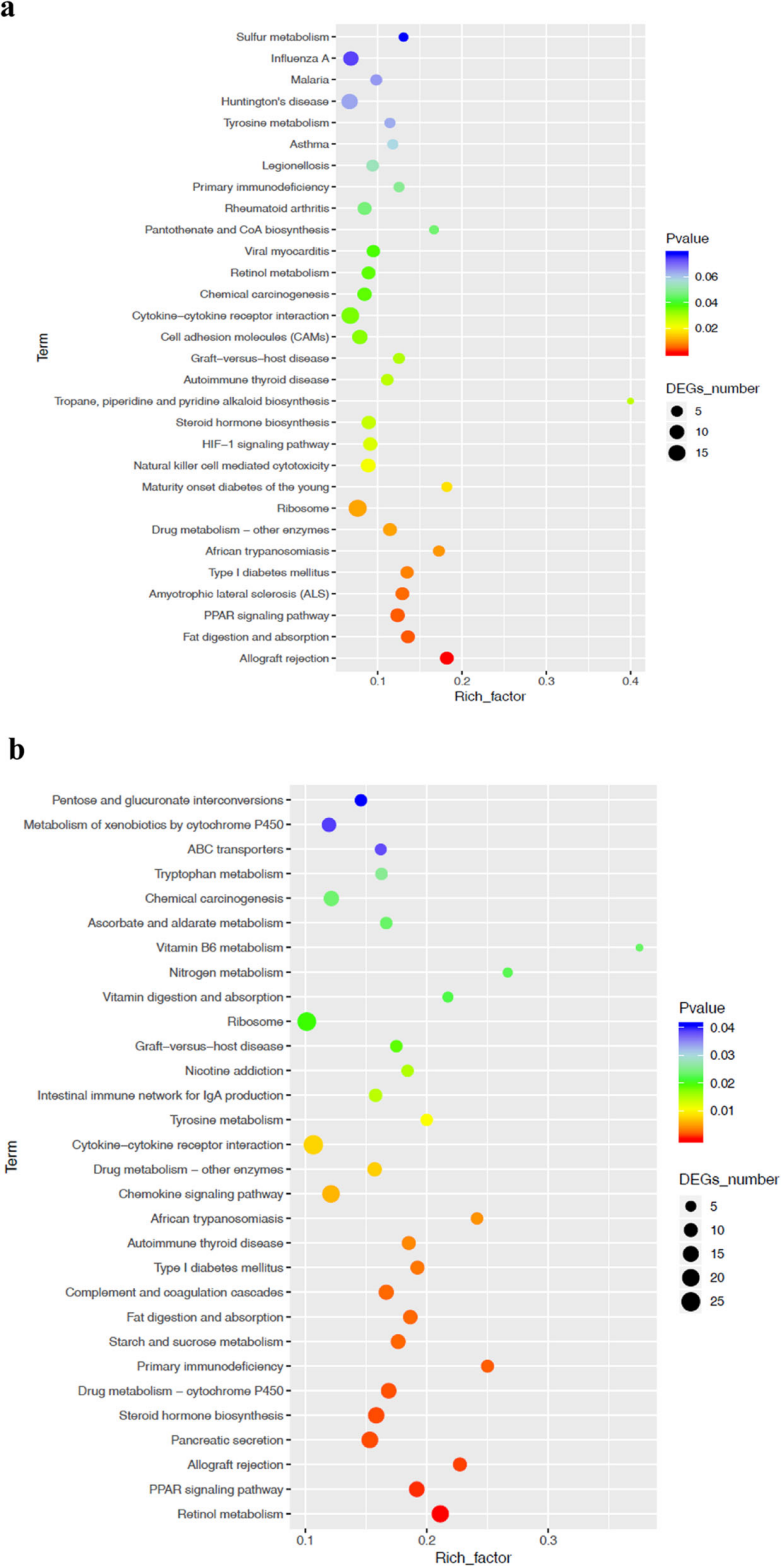
KEGG route enrichment statistics. The X-axis shows the enrichment factor; the Y-axis corresponds to the KEGG pathway. The color of the dot represents the q value, and the dot size represents the number of differentially expressed genes (DEGs) mapped to the reference path. a, b, c represent statistics of the KEGG pathway enrichment of the DEGs of the HS vs. CON, HSM vs. CON, and HSM vs. HS groups, respectively

To better understand changes in mucosa, transcriptome sequencing and differentially expressed gene identification were performed (Supplementary Table 3). The results showed that differentially expressed genes were rich on several KEGG pathways, especially the allograft rejection pathway and the fat digestion and absorption pathway **(**Fig. 4**,** *P* < 0.05). The main characteristic of the cecum exposed to HS is inflammatory immune response indicated by elevated gene expression such as *IFNG*, *IGGC*, and *CD40LG* and an element of the major histocompatibility complex (MHC) in the HS group compared to the CON group Table 1, *P* < 0.05). MOLP reduced *IFNG*, *PRF1*, granzyme B, *IL-10*, and *CD86*, and most elements of the MHC were also reduced, in the HSM group compared to the HS group (Table 1, *P* < 0.05).. Metabolic responses of cecum mucosa were also significantly changed in HS along with increased gene expression of *APOA4*, *APOA1*, *FABP1*, *FABP2*, *MTTP*, *LOC100344166*, *LOC100343913*, and *PLA2G12B*, and decreased *LOC100339512* and *PLA2G2F*. MOLP increased *FABP2*, *NPC1L1*, *ABCG8*, and *PLA2G1B*, and decreased *LOC100343913*, *PLA2G12B*, and *LOC100359181* (Table 2,  *P* < 0.05).

**Table 1:** Effect of HS and MOLP supplementation on the log fold change (logFC) of differentially expressed genes (DEGs) in pairwise comparison (HS vs. CON, HSM vs. CON and HSM vs. HS) that encode proteins in allograft rejection pathway

	Gene symbol	HS vs. CON	*P*-value	HSM vs. CON	*P-value*	*P-value*	Gene name/Predicted function
Inflammatory factor	*IFNG*	3.97	< 0.0001			0.000223	Interferon gamma precursor
	*IL12B*	−3.33	< 0.0001	−4.57	< 0.0001		Interleukin-12 subunit beta
	*IGGC*	1.81	< 0.0001	1.35	< 0.0001		Ig gamma chain C region
	*CD40LG*	11.16	0.004	10.92	0.004		CD40 ligand
	*PRF1*			−1.01	< 0.0001	< 0.0001	Perforin-1
	*LOC100101600* (granzyme H)	−2.44	< 0.0001	−3.02	< 0.0001		Granzyme H
	*LOC100101600* (granzyme B)					< 0.0001	Duodenase-1
	*IL10*					0.0014	Interleukin-10 isoform X1
	*CD86*			−1.72	0.0004	0.0055	T-lymphocyte activation antigen CD86 precursor
MHC (major histocompatibility complex)	*RLA-DMB*					< 0.0001	Histocompatibility antigen DM heterodimer light chain-like precursor
	*LOC100351416*			−1.18	< 0.0001	< 0.0001	HLA class II histocompatibility antigen, DO beta chain isoform X2
	*LOC100351163*	1.44	< 0.0001			< 0.0001	HLA class II histocompatibility antigen, DQ beta 1 chain isoform X2
	*LOC100343144*	1.12	< 0.0001			< 0.0001	SLA class II histocompatibility antigen, DQ haplotype D alpha chain isoform X2
	*RLA-DRB1*					< 0.0001	HLA class II histocompatibility antigen, DRB1–4 beta chain
	*LOC100349667*			−1.12	0.021		HLA class II histocompatibility antigen, DRB1–1 beta chain isoform X1
	*RLA-A3*					< 0.0001	MHC class I antigen-like isoform X1
	*LOC100349247*			−1.36	< 0.0001	< 0.0001	HLA class I histocompatibility antigen, B-7 alpha chain isoform X1
	*LOC100328967*					< 0.0001	RLA class I histocompatibility antigen, alpha chain 19–1 precursor
	*LOC100349085*			−1.97	< 0.0001	< 0.0001	RLA class I histocompatibility antigen, alpha chain 11/11
	*LOC100338822*	1.83	< 0.0001	1.38	< 0.0001		RLA class I histocompatibility antigen, alpha chain 11/11

It may be noted that in pairwise comparison, first group (e.g. in HS vs. CON comparison, the HS group) represents the up- or down-regulated DEGs as compared to other group when logFC value is positive or negative respectively

**Table 2:** The effect of HS and MOLP supplementation on the logfold change (logFC) of differentially expressed genes (DEGs) in pairwise comparison (HS vs. CON, HSM vs. CON and HSM vs. HS) that encode proteins in fat digestion and absorption pathway

Gene name	HS vs CON	*P*-value	HSM vs CON	*P-value*	HSM vs. HS	*P-value*	Predicted function
*APOA4*	15.69	< 0.0001	16.48	< 0.0001			Apolipoprotein A-IV precursor
*APOA1*	4.36	< 0.0001	5.01				Apolipoprotein A-I preproprotein
*FABP1*	13.49	< 0.0001	14.14	< 0.0001			Fatty acid-binding protein, liver
*FABP2*	2.62	< 0.0001	5.01	< 0.0001	2.39	< 0.0001	Fatty acid-binding protein, intestinal
*MTTP*	1.29	< 0.0001	1.32	< 0.0001			Microsomal triglyceride transfer protein large subunit
*LOC100344166*	8.89	< 0.0001	9.97	< 0.0001			Phospholipase A2, membrane associated-like
*LOC100339512*	−1.71	0.006	−2.69	< 0.0001			Pancreatic triacylglycerol lipase-like
*LOC100343913*	1.84	< 0.0001			−1.28	< 0.0001	Phospholipase A2, membrane-associated
*PLA2G2F*	−11.75	0.015	−11.75	0.015			Group IIF secretory phospholipase A2
*PLA2G12B*	2.06	0.012			1.35	0.016	Group XIIB secretory phospholipase A2-like protein isoform X2
*PLA2G5*			−1.83	< 0.0001			Calcium-dependent phospholipase A2
*NPC1L1*			12.80	< 0.0001	5.81	< 0.0001	Niemann-Pick C1-like protein 1 precursor
*APOB*			1.04	< 0.0001			Apolipoprotein B
*ABCG8*			9.84	0.015	9.84	0.008	ATP-binding cassette sub-family G member 8 isoform X2
*LOC100359181*			−2.51	< 0.0001	−1.45	< 0.0001	2-acylglycerol O-acyltransferase 2-B
*PLA2G1B*					1.60	< 0.0001	Phospholipase A2

It may be noted that in pairwise comparison, first group (e.g. in HS vs. CON comparison, the HS group) represents the up- or down-regulated DEGs as compared to other group when logFC value is positive or negative respectively

### Effect of MOLP on cecal microbiota and short chain fatty acids (SCFAs)

The statistical estimates of α-diversity from each sample at a genetic distance of 3%, presented in Supplementary Table 4, revealed no effects on any indices due to HS and dietary intervention, including the number of reads, OTUs, richness estimators (ACE and Chao1), and diversity indices (Shannon and Simpson) (Supplementary Table 4). PCA analysis on microbiota composition showed no distinction among groups (Supplementary Fig. 1). There was also no difference in the relative abundance of microbial genera among groups except for Papillibacter which was significantly higher (*P*= 0.018) in HSM group as compared to other groups (Supplementary Table 5).

**Fig. 5:**
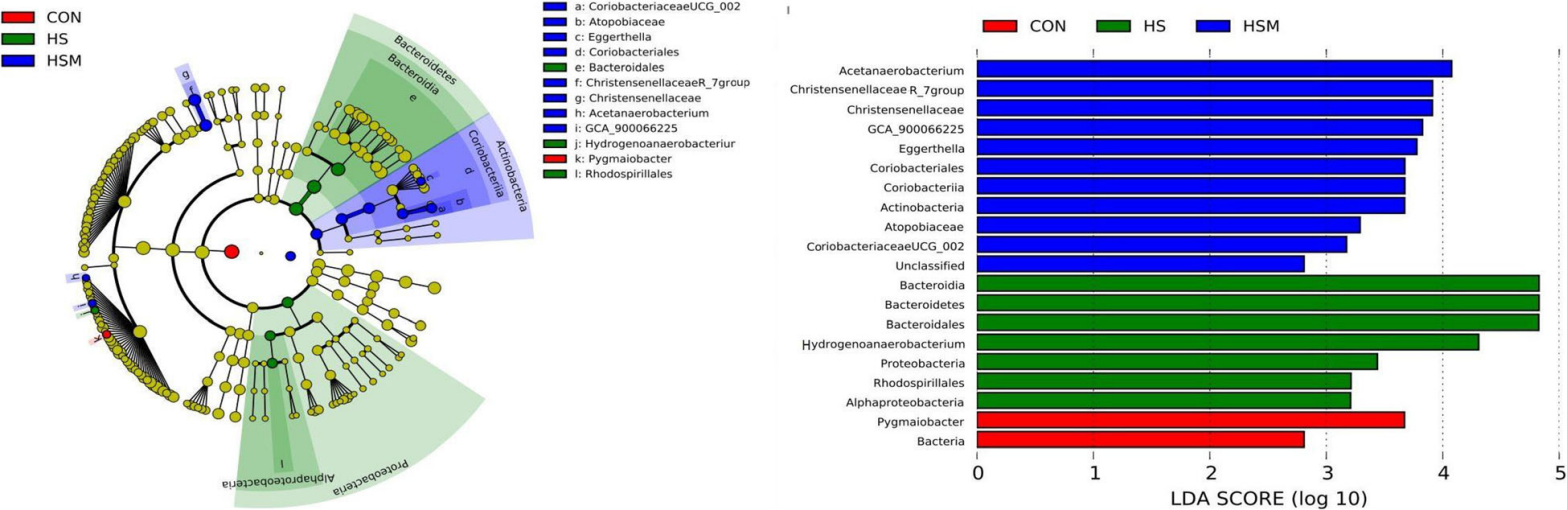
Differential analysis of microbiota community within groups i.e. CON, HS and HSM (*n* = 7 per group). Linear discrimination analysis (LDA) coupled with effect size (LEfSe) was used to identify the most differentially abundant taxa among the three groups. Only the results meeting an LDA significant threshold of > 3 were shown

Bacterial taxa (with a relative abundance of > 1% in any of the groups) were subjected to taxonomic composition analysis as previously described [27, 28]. At the phylum level, Firmicutes and Bacteroidetes were the most predominant phyla in the cecal digesta of rabbits, with a total relative abundance of around 90%. In descending order, the predominance of phyla was ranked as follows: Firmicutes > Bacteroidetes > Verrucomicrobia > Proteobacteria > Actinobacteria **(**Table 3**)**. The relative abundance of Actinobacteria and Proteobacteria were significantly different between the groups (Table 3, *P* < 0.05), whereas no significant change in relative abundance was observed at the genus level except for Papillibacter (Supplementary Table 5). LDA effect size (LEfSe) analysis further revealed increased relative abundance of Actinobacteria in the HSM group and increased abundance of Proteobacteria and Bacteroidetes in HS group (Fig. 5*P* < 0.05). Total SCFA concentrations remained unchanged, whereas acetic acid and butyric acid were significantly higher in the HSM group as compared to the CON and HS groups (Table 4, *P* < 0.05).

**Table 3:** Effect of HS and MOLP supplementation on microbiota composition in cecal digesta of NZW rabbits at phylum level (relative abundance of top 5 phyla)

Items	CON	HS	HSM	SEM	*P*-value
Firmicutes	57.83	59.18	66.15	2.31	0.299
Bacteroidetes	30.61	29.10	21.04	2.35	0.206
Verrucomicrobia	9.24	7.92	8.27	1.50	0.943
Proteobacteria	0.56^b^	1.05^a^	0.62^b^	0.07	0.002
Actinobacteria	0.36^b^	0.54^b^	1.21^a^	0.15	0.037

*MOLP Moringa oleifera* leaf powder; *CON* control; *HS* Heat stress; *HSM* Heat stress with *Moringa* supplementation. All data is presented as mean values ± standard error of mean (SEM). ^a,b^ Means in rows with different superscript letters are significantly different (*P <* 0.05)

**Table 4:** Effect of HS and MOLP supplementation on the concentrations of short chain fatty acids (SCFAs) in cecal digesta in heat-stressed NZW rabbits (*n* = 7 per group)

Item, mmol/L	CON	HS	HSM	SEM	*P*-value
Acetic acid	25.3^a^	21.4^b^	38.0^a^	2.97	0.004
Propionic acid	9.50	9.60	5.80	1.33	0.47
Butyric acid	9.20^b^	9.00^b^	11.5^a^	1.19	0.014
Iso-butyric acid	0.90	1.70	1.80	0.27	0.49
Valeric acid	1.10	1.00	0.90	0.67	0.91
Iso-valeric acid	0.30	0.30	0.50	0.07	0.39
Total SCFAs	45.3	41.2	53.5	4.67	0.28

*MOLP Moringa oleifera* leaf powder; *CON* Control; *HS* Heat stress; *HSM* Heat stress with MOLP supplementation. All data is presented as mean values ± standard error of mean (SEM). ^a,b^ Means in rows with different superscript letters are significantly different (*P <* 0.05)

## Discussion

HS is one of the largest impediments to efficient animal agriculture, resulting in hampered growth performance, altered carcass composition, and reduced fertility in rabbits [6, 29–32]. Traditionally, the negative impacts of HS on animal performance were attributed to associated decreases in feed intake, likely as a strategy to reduce metabolic heat production [7]. Similar results were obtained in our previous study demonstrating significantly reduced ADFI, ADG, and FCR in heat stressed rabbits [12]. Whereas *Moringa* supplementation significantly improved these traits. As an activated immune system (such that under HS) requires a substantial amount of energy, nutrients (specifically glucose and amino acids) are diverted from anabolic processes to support the immune response and thereby sustain growth and reproduction [33, 34]. In other words, supporting the activated immune system becomes a priority, and the hierarchy of nutrient partitioning is coordinated to ensure survival, whereas the promotion of economically important phenotypes is deemphasized.

Gastrointestinal tract is a major organ affected by heat stress [35]. In actuality, the many negative consequences of HS might be mediated by hyperthermia’s adverse effects on the intestinal epithelial barrier [8]. Liu et al. [36, 37] demonstrated that diurnal heat stress caused significant increase in permeability of small intestine as well as decrease in growth performance of heat stressed pigs compared with controlled ones. Similarly using a 3 day diurnal heat stress model on nursery-grower pig performance, intestinal integrity and endotoxemia; Gabler et al. [35] concluded that HS mediated reduction in ileum integrity and higher circulating LPS, altered blood metabolites and inflammation profiles might result in reduce performance of pigs over summer months. Moreover, dietary antioxidants (vitamin E & Se) have been described to mitigate the impacts of HS on intestinal barrier integrity, associated with a reduction in oxidative stress [37]. Keeping in view the results of current study, we can conclude that disruption of the intestinal epithelial barrier may be the indirect reason for the decreased growth performance and reduced organ index of cecum under HS.

HS causes a diversion of blood flow from the splanchnic bed to the periphery in a thermoregulatory effort to increase radiant heat loss. This altered cardiovascular event reduces blood flow and nutrient delivery to the gastrointestinal tract, resulting in local hypoxia, free radical production, and compromised intestinal architecture [38, 39]. The reactive oxygen species (ROS) readily attack and induce oxidative damage to various biomolecules, including proteins, lipids, lipoproteins, and DNA [40]. Lipid per oxidation (LPO) has been used as a reliable marker of oxidative stress, both *in vitro* and *in vivo* [41]. Antioxidant enzymes, such as SOD, catalase, and glutathione peroxidase (GPx), help to reduce oxidative stress in various parts of the cell [42]. These antioxidants may mediate their effect by directly reacting with ROS, quenching them and/or chelating the catalytic metal ions [43]. Herbs like *Moringa oleifera* contain free radical scavengers, such as polyphenols, flavonoids, and phenolic compounds [44–47]. Some polyphenol metabolites may have prebiotic, anti-inflammatory, anti-oxidative, anti-carcinogenic, and anti-microbial properties [44–47]. The present study demonstrated a significant reduction in cecal mucosal MDA content in rabbits fed a *Moringa oleifera*-supplemented diet. Whereas higher MDA levels and SOD activity in the HS group suggested lipid peroxidation and oxidative stress under HS environment respectively. This reduction could suggest a decrease in LPO and the potential of *Moringa oleifera* to prevent the formation of excessive free radicals by acting as chain-breaking peroxyl-radical scavengers and to protect low-density lipoproteins from oxidation [48]. Thus, the results of the current study highlight the antioxidant effects rendered by MOLP supplementation under oxidative stress conditions.

HS causes intestinal hyperpermeability—consequently, a dysfunctional intestinal barrier allows for the translocation of both dietary and microbial antigens (e.g., LPS from gram-negative bacteria) from the lumen through the basolateral membrane, eliciting local and systemic inflammatory responses [38, 39]. Accordingly, increased circulatory pro-inflammatory cytokines (e.g., TNFα, IL-1α, IL-1β, IL-6) have been reported in different HS models, presumably linked to increased circulating LPS [49–53]. In support of this, higher cecal mucosa levels of LPS, TNFα, IL-1α, IL-1β, and MPO due to HS, and lower levels due to MOLP, were observed. Further, based on the transcriptome analysis of cecal mucosa, the main characteristic of the cecum exposed to HS is inflammatory immune response indicated by elevated gene expression such as *IFNG*, *IGGC*, *CD40LG*, and an ingredient of the MHC compared to the CON group. MOLP down-regulated *IFNG*, *PRF1*, granzyme B, *IL-10*, and *CD86*, as well as most of the MHC, compared to the HS group. Up-regulation of *IL-10* (secreted by regulatory T-lymphocytes, monocytes and macrophages) in cecal mucosa of HS group seems to inhibit the enhanced production of pro-inflammatory cytokines such as *TNF-α* and *IFNG* as reported previously [54]. Under inflammatory conditions, up-regulated *IFNG* (a potent pro-inflammatory cytokine) mediates both apoptotic and apoptosis-independent loss of intestinal epithelial integrity [55]. Downregulation of *INFG* in HSM group suggests that the *Moringa* may be exerting an anti-inflammatory immune response. Results are consistent with other studies indicating down-regulation of pro-inflammatory cytokines (e.g. *INFG*) with oral application of Lactobacillus-based products in mice [56, 57]. HS affected the functions of immunoglobulins, which play an important role in immune regulation and mucosal defense. IgA protects the mucosal surfaces of the intestine by preventing the entry, binding, and colonization of toxins and pathogens in both animal and human models [58]. The concentration of IgA was significantly lower in the HS group, whereas no difference was observed in the CON and HSM groups. This finding suggests that dietary *Moringa oleifera* was effective in eliciting a humoral immune response in rabbits under HS. Combined with the findings of our previous study, it can be suggested that HS causes an inflammatory response in both the jejunum [12] and cecum.

Despite marked reductions in feed intake and body weight loss, HS attenuates lipid mobilization in rodents [59] and ruminants [60–63]. Additionally, increased lipid carcass deposition occurs in heat-stressed growing animals [64], especially when compared to pair-fed thermoneutral controls [65, 66]. In our study, the results showed that both the HS and HSM groups had increased gene expression of fat digestion and absorption pathway, including *APOA1*, *FABP1*, *FABP2*, *MTTP*, and *LOC100344166*, compared to the CON group. *Moringa* has been described to possess anti-hyperlipidemia properties [67]. So, similar expression pattern of genes related to fat digestion and absorption pathway in HS (CON vs. HS) and HSM (CON vs. HSM) group still remains to be made clear for anti-hyperlipidemia effects of *Moringa* under HS.

HS and *Moringa oleifera* supplementation had a significant effect on the cecal microbial population at the phylum level assessed in this study. Firmicutes and Bacteroidetes were found to be the two most predominant phyla, followed by Verrucomicrobia, in accordance with previous studies [68], further followed by Proteobacteria and Actinobacteria. It has been reported that Firmicutes, Bacteroidetes, Proteobacteria, and Verrucomicrobia (90.5–97.7%) play an important role in the cecum, duodenum, and jejunum of growing rabbits [69]. In our study, dominant phyla altered by HS were Bacteroidetes (Fig. 5) and Proteobacteria (Table 3, Fig. 5), both being higher in abundance, whereas *Moringa oleifera* supplementation increased the abundance of Actinobacteria (Table 3). Whereas, at genus level, higher abundance of Papillibacter was found in HSM group (Supplementary Table 3). Shin et al. [70] proposed an increased prevalence of Proteobacteria as a potential diagnostic signature of dysbiosis and intestinal inflammation. Consistent findings have commonly supported an increased prevalence of Proteobacteria to the concept of dysbiosis during metabolic disorders [71, 72]. Likewise, a Chinese pilot study indicated dysbiosis of gut microbiota (significant enrichment of Proteobacteria and Bacilli) along with reduction in short chain fatty acids in patients with Encephalitis [73]. Moreover, gut dysbiosis with an excessive growth of Prevotellaceae and Bacteroidetes has been described in NLRP3 deficient mice under normal conditions [74].

Genus Papillibacter has been found naturally in Shea cake fed anaerobic digester, anaerobic sludge (from the pit of slaughter-houses) and environmental samples [75]. However, the metabolic and functional significance in the ruminal or cecal ecosystem is currently unknown. The only species of this genus ‘*Papillibacter cinnaminivorans*’ is phylogenetically related to the flavonoid ring-cleaving *Clostridium orbiscindens* anaerobe which is present in gastrointestinal tract of humans [75]. This may explain the reason why Polyphenol rich *Moringa* supplementation increased the abundance of Papillibacter in HSM group.

One of the cecum’s main functions is bacterial fermentation of indigestible polysaccharides to produce SCFAs, which can be utilized by the host’s epithelial cells. SCFAs can contribute 20–30% of the caloric requirements in rabbits [76]. Acetate, butyrate, and propionate are end products of the microbial fermentation of macronutrients. As epithelial cells utilize butyrate as their principal energy source, a ready source in the mucus is likely to support early epithelial maturation and growth [77]. SCFAs play an important role in gut physiology. Butyrate, in particular, is widely regarded as health-promoting. As stated, butyrate has been demonstrated as a mediator of anti-inflammatory responses, in the maintenance of the intestinal barrier integrity and in the protection against oxidative stress [78]. It can be stated that the higher butyrate levels indicate self-protection of the gastrointestinal tract under the *Moringa oleifera* supplementation regimen for HS. Although the results from current study warrant further investigation due to the limited number of rabbits used, yet the present study provided a logical basis for the use of *Moringa oleifera* in rabbit husbandry under HS conditions.

## Conclusion

It can be summarized that HS results in change in redox status while *Moringa* supplementation reduced oxidative stress in mucosal tissue by reducing protein contents of MDA, SOD and LDH. Heat stress also resulted in dysbiosis of cecal microbiota and reduced integrity of cecal mucosa as evident by higher abundance of Proteobacteria and enhanced contents of LPS respectively, in HS group. Increased inflammatory immune response occurred in HS by elevated expression of pro-inflammatory genes such as IFNG and protein expression of inflammatory markers such as TNF-α, IL-1α, and IL-1β along with higher abundance of putatively pro-inflammatory gram negative Bacteroidetes. Whereas, *Moringa* supplementation reversed these conditions. *Moringa* supplementation also improved ADG and organ index of cecum. Moreover, higher abundance of genus *Papillibacter* and enhanced levels of microbial metabolite ‘Butyrate’ in HSM group demonstrate the ability of polyphenol rich *Moringa* to favorably module cecal microbial ecosystem. Concluding, *Moringa* supplementation modulated mucosal immune response and microbial ecosystem of cecum under HS by the mechanism of reduced oxidative stress and inflammatory markers along with reduction in the relative abundance of bacteria linked with dysbiosis.

## Supplementary Information

**Additional file 1: Table S1.** Nutrient composition per 100 g of *Moringa oleifera* leaf powder (MOLP).

**Additional file 2: Table S2.** Effect of heat stress and MOLP supplementation on productive performance of New Zealand White rabbits.

**Additional file 3: Table S3.** Summary of the sample comparison results of differentially expressed genes (DEGs).

**Additional file 4: Table S4.** Summary of Illumina Miseq sequence data and statistical analysis of the bacterial diversity in the cecum of CON, HS, and HSM groups (*n* = 7 per group).

**Additional file 5: Table S5.** Effect of HS and MOLP supplementation on the relative abundance of microbial genera (with percentages greater than 0.5% in any of groups) in cecum of NZW rabbits (*n* = 7 per group).

**Additional file 6: Figure S1.** Unweighted UniFrac Principal component analysis (PCA) of microbiota composition in cecum based on operational taxonomic unit (OUT) data (*n* = 7 per gorup). CON: control group; HS: heat stress group; HSM: heat stress with MOLP supplementation group; MOLP: *Moringa oleifera* leaf powder.

## Data Availability

The datasets used and analyzed during the current study are available from the corresponding author on reasonable request.

## Abbreviations

ADG: Average daily gain
ADFI: Average daily feed intake
FCR: Feed conversion ratio
CAT: Catalase
CON: Control
DEGs: Differentially expressed genes
FDR: False discovery rate
HS: Heat stress
HSM: HS supplemented orally with MOLP
GST: Glutathione S-transferase
IL: Interleukin
IL-1α: Interleukin-1α
IL-1β: Interleukin-1β
IL-6: Interleukin-6
LDH: Lactate dehydrogenase
LEfSe: LDA effect size
LPS: Lipopolysaccharide
MDA: Malonaldehyde
MOLP: *Moringa oleifera* leaf powder
MPO: Myeloperoxidase
NZW: New Zealand White
SIgA: Secretory immunoglobulin A
SCFAs: Short chain fatty acids
OTUs: Operational taxonomic units
SOD: Superoxide dismutase
TNF-α: Tumor necrosis factor alpha
