# Supplementary material for: Oral administration of Moringa oleifera leaf powder relieves oxidative stress, modulates mucosal immune response and cecal microbiota after exposure to heat stress in New Zealand White rabbits
Source: J Anim Sci Biotechnol. 2021 May 12;12:66. doi: 10.1186/s40104-021-00586-y (PMC8114525; doi:10.1186/s40104-021-00586-y)
Supplement: Supplementary file 1 — Additional file 1: Table S1. Nutrient composition per 100 g of Moringa oleifera leaf powder (MOLP). [file 40104_2021_586_MOESM1_ESM.doc]

**Supplementary Table 1**

Nutrient composition per 100g of *Moringa oleifera* leaf powder (MOLP)

| Amount Per 100g | Unit | Content |
| --- | --- | --- |
| Water | % | 6.30 |
| Energy | KJ | 1191.00 |
| Protein | g | 27.50a |
| Fat | g | 8.70 |
| [Carbohydrate](javascript:;) | g | 47.47 |
| Total dietary fiber | g | 23.77 |
| [Calcium](javascript:;) | mg | 2357.03 |
| [Magnesium](javascript:;) | mg | 395.03 |
| [Phosphorus](javascript:;) | mg | 280.80 |
| [Potassium](javascript:;) | mg | 1759.37 |
| [Selenium](javascript:;) | mg | 13.10 |
| [Copper](javascript:;) | mg | 0.50 |
| [Sulphur](javascript:;) | mg | 870 |
| Zinc | mg | 2.78 |
| Iron | mg | 13.54 |
| sodium | mg | 416.47 |
| [Vitamin](javascript:;) A | mg | 41870 |
| [Vitamin](javascript:;) B_1_ | mg | 0.14 |
| [Vitamin](javascript:;) B_2_ | mg | 0.99 |
| [Vitamin](javascript:;) B_3_ | mg | 10.74 |
| [Vitamin](javascript:;) C | mg | 73.90 |
| [Vitamin](javascript:;) E | mg | 155.67 |
