# Supplementary material for: Oral administration of Moringa oleifera leaf powder relieves oxidative stress, modulates mucosal immune response and cecal microbiota after exposure to heat stress in New Zealand White rabbits
Source: J Anim Sci Biotechnol. 2021 May 12;12:66. doi: 10.1186/s40104-021-00586-y (PMC8114525; doi:10.1186/s40104-021-00586-y)
Supplement: Supplementary file 2 — Additional file 2: Table S2. Effect of heat stress and MOLP supplementation on productive performance of New Zealand White rabbits. [file 40104_2021_586_MOESM2_ESM.doc]

**Supplementary Table 2** Effect of heat stress and MOLP supplementation on productive performance of New Zealand White rabbits*

| Item | Treatments | | | *p* value |
| --- | --- | --- | --- | --- |
|  | CON | HS | HSM |  |
| IBW | 3291 ± 119 | 3336 ± 281 | 3327 ± 115 | 0.958 |
| FBW | 3857 ± 119 | 3577 ± 96.6 | 3819 ± 93.3 | 0.153 |
| ADG (g) | 18.2 ± 0.57^a^ | 11.2 ± 0.42^c^ | 14.1 ± 0.61^b^ | < 0.001 |
| ADFI (g) | 107 ± 3.96^a^ | 74.2 ± 3.11^c^ | 85.1 ± 1.91^b^ | < 0.001 |
| FCR | 5.85 ± 0.085^b^ | 6.78 ± 0.11^a^ | 6.03 ± 0.29^b^ | 0.006 |

Note. All data is shown as mean values ± standard error of the mean (SEM) for 4 weeks.

MOLP: *Moringa oleifera* leaf powder; CON: control treatment; HS: heat stress; HSM: heat stress with MOLP supplementation; IBW: initial body weight; FBW: final body weight; ADG: average daily gain; ADFI: average daily feed intake; FCR: feed conversion ratio.

^a,b,c^ Means within a row with different superscript letters are signiﬁcantly different (*P <* 0.05).

* Khalid AR, Yasoob TB, Zhang Z, Yu D, Feng J, Zhu X, et al. Supplementation of Moringa oleifera leaf powder orally improved productive performance by enhancing the intestinal health in rabbits under chronic heat stress. J Therm Biol [Internet]. Elsevier Ltd; 2020;93:102680. Available from: https://doi.org/10.1016/j.jtherbio.2020.102680
