# Supplementary material for: Oral administration of Moringa oleifera leaf powder relieves oxidative stress, modulates mucosal immune response and cecal microbiota after exposure to heat stress in New Zealand White rabbits
Source: J Anim Sci Biotechnol. 2021 May 12;12:66. doi: 10.1186/s40104-021-00586-y (PMC8114525; doi:10.1186/s40104-021-00586-y)
Supplement: Supplementary file 3 — Additional file 3: Table S3. Summary of the sample comparison results of differentially expressed genes (DEGs). [file 40104_2021_586_MOESM3_ESM.doc]

**Supplementary Table 3.** Summary of the sample comparison results of differentially expressed genes (DEGs).

| Sample | All genes | Up-regulated | Down-regulated |
| --- | --- | --- | --- |
| CON_vs_HS | 802 | 228 | 574 |
| CON_vs_HSM | 1382 | 235 | 1147 |
| HSM_vs_HS | 807 | 587 | 220 |

The screening criterion to select the differential expressed genes between groups was used when threshold fold change |log2FC|≥ = 1 & FDR < 0.05
