# Supplementary material for: Oral administration of Moringa oleifera leaf powder relieves oxidative stress, modulates mucosal immune response and cecal microbiota after exposure to heat stress in New Zealand White rabbits
Source: J Anim Sci Biotechnol. 2021 May 12;12:66. doi: 10.1186/s40104-021-00586-y (PMC8114525; doi:10.1186/s40104-021-00586-y)
Supplement: Supplementary file 4 — Additional file 4: Table S4. Summary of Illumina Miseq sequence data and statistical analysis of the bacterial diversity in the cecum of CON, HS, and HSM groups (n = 7 per group). [file 40104_2021_586_MOESM4_ESM.doc]

**Supplementary Table 4. Summary of Illumina Miseq sequence data and statistical analysis of the bacterial diversity in the cecum of CON, HS, and HSM groups (*n* = 7).**

| Items | CON | HS | HSM | SEM | *P*-value |
| --- | --- | --- | --- | --- | --- |
| Reads | 40666 | 38968 | 37869 | 1174 | 0.642 |
| OTU | 829 | 1003 | 923 | 42 | 0.254 |
| Chao | 973 | 1142 | 1084 | 42 | 0.264 |
| Coverage | 0.9958 | 0.9953 | 0.9952 | 0.0002 | 0.544 |
| Shannon | 4.06 | 4.91 | 4.86 | 0.22 | 0.233 |
| Simpson | 0.0395 | 0.0290 | 0.0334 | 0.0046 | 0.671 |

CON: control group; HS: heat stress group; HSM: heat stress with MOLP supplementation group; MOLP: *Moringa oleifera* leaf powder. All data is shown as mean values ± standard error of the mean (SEM).
