# Supplementary material for: Oral administration of Moringa oleifera leaf powder relieves oxidative stress, modulates mucosal immune response and cecal microbiota after exposure to heat stress in New Zealand White rabbits
Source: J Anim Sci Biotechnol. 2021 May 12;12:66. doi: 10.1186/s40104-021-00586-y (PMC8114525; doi:10.1186/s40104-021-00586-y)
Supplement: Supplementary file 5 — Additional file 5: Table S5. Effect of HS and MOLP supplementation on the relative abundance of microbial genera (with percentages greater than 0.5% in any of groups) in cecum of NZW rabbits (n = 7 per group). [file 40104_2021_586_MOESM5_ESM.doc]

**Supplementary Table 5.** Effect of HS and MOLP supplementation on the relative abundance of microbial genera (with percentages greater than 0.5% in any of groups) in cecum of NZW rabbits (*n* = 7).

| Items | CON | HS | HSM | SEM | *P*-value |
| --- | --- | --- | --- | --- | --- |
| Ruminococcaceae NK4A214 group | 11.51 | 10.70 | 10.62 | 1.09 | 0.943 |
| Lachnospiraceae NK4A136 group | 8.26 | 6.96 | 10.09 | 1.43 | 0.680 |
| Akkermansia | 9.24 | 7.92 | 8.27 | 1.50 | 0.943 |
| Bacteroides | 10.13 | 10.67 | 3.86 | 1.78 | 0.220 |
| Ruminococcaceae UCG-014 | 4.11 | 6.14 | 7.53 | 0.79 | 0.224 |
| Rikenellaceae RC9 gut group | 5.93 | 5.94 | 4.92 | 1.51 | 0.955 |
| dgA-11 gut group | 3.48 | 6.30 | 4.87 | 0.76 | 0.346 |
| Muribaculaceae_norank | 6.82 | 2.25 | 4.23 | 1.18 | 0.310 |
| Clostridiales vadinBB60 group_norank | 3.36 | 3.67 | 2.74 | 0.48 | 0.734 |
| Ruminococcus 1 | 2.80 | 3.78 | 2.62 | 0.43 | 0.511 |
| Eubacteriaceae_uncultured | 2.75 | 2.36 | 2.96 | 0.52 | 0.897 |
| Fusicatenibacter | 2.02 | 2.29 | 3.56 | 0.32 | 0.113 |
| Christensenellaceae R-7 group | 2.04 | 2.13 | 3.16 | 0.27 | 0.157 |
| Ruminococcaceae V9D2013 group | 2.10 | 2.59 | 2.14 | 0.37 | 0.845 |
| Ruminococcaceae UCG-010 | 2.11 | 2.25 | 2.23 | 0.21 | 0.962 |
| Alistipes | 2.31 | 2.03 | 1.31 | 0.28 | 0.346 |
| Ruminococcaceae UCG-013 | 1.61 | 1.53 | 2.23 | 0.17 | 0.196 |
| Ruminococcaceae_uncultured | 1.59 | 2.07 | 1.68 | 0.23 | 0.699 |
| Ruminiclostridium 6 | 1.42 | 1.37 | 2.20 | 0.40 | 0.660 |
| Lachnospiraceae_uncultured | 1.12 | 1.51 | 1.22 | 0.15 | 0.558 |
| Lachnospiraceae_Unclassified | 1.25 | 0.90 | 1.23 | 0.15 | 0.569 |
| Phascolarctobacterium | 1.10 | 1.24 | 0.62 | 0.23 | 0.522 |
| Subdoligranulum | 0.66 | 0.98 | 1.12 | 0.16 | 0.510 |
| Barnesiellaceae_uncultured | 0.85 | 1.03 | 0.67 | 0.10 | 0.308 |
| Papillibacter | 0.52 | 0.39 | 1.06 | 0.11 | 0.018 |
| Ruminococcus 2 | 0.64 | 0.16 | 1.00 | 0.19 | 0.166 |
| [Eubacterium] ruminantium group | 1.06 | 0.29 | 0.35 | 0.30 | 0.554 |

CON: control group; HS: heat stress group; HSM: heat stress with MOLP supplementation group; MOLP: *Moringa oleifera* leaf powder. All data is shown as mean values ± standard error of the mean (SEM).
