# Supplementary material for: Oral administration of Moringa oleifera leaf powder relieves oxidative stress, modulates mucosal immune response and cecal microbiota after exposure to heat stress in New Zealand White rabbits
Source: J Anim Sci Biotechnol. 2021 May 12;12:66. doi: 10.1186/s40104-021-00586-y (PMC8114525; doi:10.1186/s40104-021-00586-y)
Supplement: Supplementary file 6 — Additional file 6: Figure S1. Unweighted UniFrac Principal component analysis (PCA) of microbiota composition in cecum based on operational taxonomic unit (OUT) data (n = 7 per gorup). CON: control group; HS: heat stress group; HSM: heat stress with MOLP supplementation group; MOLP: Moringa oleifera leaf powder. [file 40104_2021_586_MOESM6_ESM.doc]

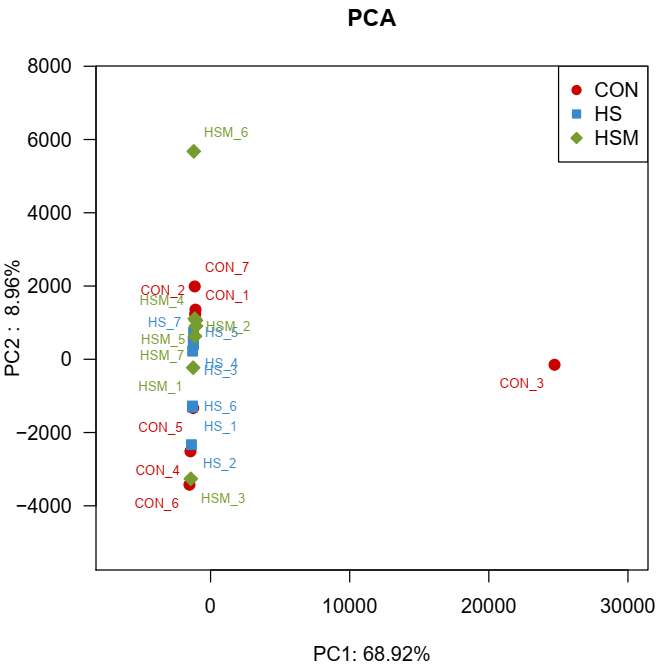


**Supplementary Figure 1.** Unweighted UniFrac Principal component analysis (PCA) of microbiota composition in cecum based on operational taxonomic unit (OUT) data (*n* = 7). CON: control group; HS: heat stress group; HSM: heat stress with MOLP supplementation group; MOLP: *Moringa oleifera* leaf powder.
